# Tyrosinase-Catalyzed Soy Protein and Tannic Acid Interaction: Effects on Structural and Rheological Properties of Complexes

**DOI:** 10.3390/gels11030195

**Published:** 2025-03-12

**Authors:** Yaqiong Pei, Lei Yuan, Wenjing Zhou, Jun Yang

**Affiliations:** College of Food Science and Technology, Wuhan Business University, Wuhan 430056, China

**Keywords:** soy protein isolate, tannic acid, tyrosinase, structure, rheological properties

## Abstract

This study investigated the structural, rheological, and microstructural properties of soy protein isolate (SPI) induced by tyrosinase-catalyzed crosslinking with tannic acid (TA) at 25 °C under neutral conditions at pH 6.5. The particle size and polydispersity index of modified SPI progressively increased with rising TA concentrations. Tyrosinase-induced polymerization significantly impacted the conformational structure of SPI, evidenced by a notable decrease in intrinsic fluorescence, a pronounced red shift, and a remarkable reduction in surface hydrophobicity. FTIR analysis further revealed that, compared to control SPI, the amide I, II, and III bands of SPI incubated with TA and tyrosinase exhibited varying degrees of red-shift or blue-shift. These observations suggested a substantial alteration in the secondary structure of SPI after incubation with TA and tyrosinase. The apparent viscosity, G′, and G″ of the modified SPI increased with higher TA concentrations, indicating that the modification of SPI by TA in the presence of tyrosinase resulted in enhanced covalent crosslinking. Microstructural observations confirmed that higher TA levels promoted the formation of denser and more uniform gel-like networks. The findings demonstrated that tyrosinase-mediated crosslinking improved the functionality of SPI, making it a promising approach for food applications.

## 1. Introduction

Soy protein isolate (SPI) has been extensively utilized as a valuable ingredient in functional vegetable protein beverages, tofu, gel bean products, and meat analogs, owing to its low cost and superior nutritional profile, which includes all essential amino acids required by the human body. Additionally, SPI is recognized for its diverse functionalities, such as fat replacement, gelling, emulsifying, and foaming capacities [1,2,3]. However, the physicochemical stability of soy protein isolate can be adversely affected by common food processing conditions, including temperature fluctuations (both heat and cold treatments), variations in ionic strength, and changes in pH. These factors may lead to coalescence, flocculation, and aggregation, thereby limiting the functionality and applicability of SPI in food systems [4,5]. Protein function is intricately linked to its structure and conformation; thus, modulating these properties can enhance functionality. The commonly used methods for protein modification can be categorized into three main types: physical methods (such as ultrasonic treatment, heat treatment, high-pressure homogenization, and pulsed electric field) [6], chemical methods (including glycosylation, glutaraldehyde-induced cross-linking, and free radical grafting) [7], and biological methods (primarily enzymatic catalytic cross-linking) [8,9]. Compared to the high energy consumption associated with physical methods and the complex derivatives and reagent residues generated by chemical methods [10], enzymatic modification offers distinct advantages due to its efficiency and milder reaction conditions. Recent studies have drawn significant attention to protein–polyphenol covalent interactions through oxidation [11,12,13,14] and enzyme-catalyzed reactions [15,16,17,18,19,20] involving phenolic compounds, which generate radicals or quinones capable of reacting with the amino acid side chains of proteins. Current studies predominantly focus on specific compounds such as rosmarinic acid, epigallocatechin gallate, ferulic acid, gallic acid, and chlorogenic acid. Among polyphenols, tannic acid emerges as a promising candidate for enzymatic modifications due to its remarkable ability to form stable protein complexes, making it an ideal choice for enhancing protein functionality.

Tannic acid (TA), a polyphenol abundantly found in plants, serves as an essential commercial raw material and is widely utilized in various industries, such as leather tanning, coatings, adhesives, surgery, pharmaceuticals, and food processing [21]. Due to its multiple catechol and pyrogallol aromatic ring structures, along with a high number of hydroxyl groups and other functional groups such as carboxyls, enable TA to form strong complexes with proteins and other macromolecules [22]. Furthermore, TA exhibits excellent antimicrobial, anti-inflammatory, anticancer, and antioxidant properties [23], positioning it as a promising candidate for safe and healthy food ingredients. The interaction of TA with proteins can stabilize their structures and enable them to perform their intrinsic functions, which is significant for broadening the application fields of proteins. The influence of both non-covalent interactions between TA and proteins [10,23,24,25,26,27,28,29,30] and chemical covalent interactions between TA and proteins [12,31,32,33] on the structural and functional properties of proteins has been extensively studied. However, to the best of our knowledge, enzymatic-catalyzed covalent interactions between TA and proteins are relatively rare. Notably, the Vate team reported that the combination of squid ink tyrosinase with tannic acid can significantly enhance surimi gel performance [34,35]. Research on the interaction between TA and soy protein isolates through enzymatic methods to enhance their structural characteristics and functional properties remains unexplored.

In the current study, the impact of tannic acid (TA) modification on the structure and of soy protein isolate (SPI) was evaluated by comparing modified SPI samples with and without the addition of tyrosinase (TYRase). Surface hydrophobicity, intrinsic fluorescence spectroscopy and Fourier transform infrared spectroscopy (FTIR) were employed to investigate the structural changes in modified SPI induced by TA and TYRase. Additionally, particle size, zeta-potential, apparent viscosity, viscoelastic properties (G′ and G″), and the microstructure of modified SPI were characterized. This study aims to explore how TYRase-catalyzed crosslinking with tannic acid can impact the structural and functional properties of SPI, offering an alternative approach to improving protein functionality in food systems. The findings will provide insights into the mechanisms of SPI-TA interactions and their potential applications in food science and technology. Future research should further analyze the interfacial characteristics, nutritional properties, and practical applications of SPI-TA covalent complexes catalyzed by enzymes, to enhance the effectiveness of SPI in food processing.

## 2. Results and Discussion

### 2.1. The Particle Size and Zeta-Potential

**Figure 1:**
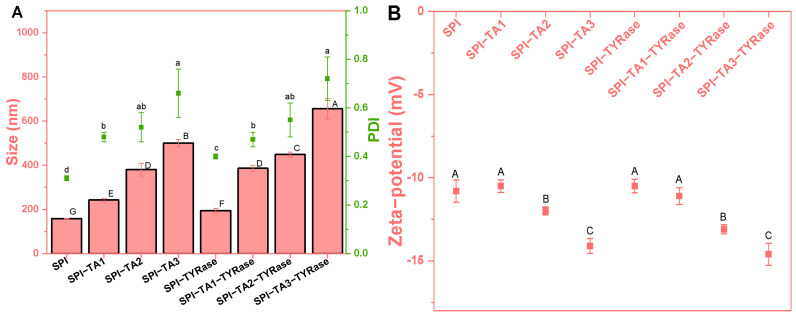
Particle size (bar chart) and polydispersity index (scatter chart) (**A**) and zeta-potential (**B**) of SPI incubated with tannic acid (SPI-TA) and with tannic acid and tyrosinase (SPI-TA-TYRase) at varying tannic acid concentrations (TA1 0.01%, TA2 0.03%, TA3 0.05%) after a 24 h incubation at room temperature (pH 6.5). Different lower or upper case letters indicate significant differences in the results (*p* < 0.05).

Initially, the effects of tyrosinase (TYRase) and tannic acid (TA) concentration (ranging from 0 to 0.05% *w*/*w*) on the particle size, polydispersity index (PDI) and zeta-potential of modified SPI at a concentration of 0.5% *w*/*w* were measured (Figure 1). The presence of TYRase and TA generally led to an increase in protein particle size, with a more pronounced effect observed as the concentration of TA increased. At a TA concentration of 0.05%, the particle sizes measured 500.5 nm in the absence of TYRase and 655.8 nm in its presence. Furthermore, the PDI of the sample increased with rising TA concentrations. Notably, when the TA concentration reached 0.05%, the PDI value for SPI-TA3 was 0.66 ± 0.10, indicating a significant degree of SPI aggregation. These phenomena could be attributed to the ability of TA to connect SPI intramolecularly and intermolecularly in the absence of tyrosinase through both non-covalent interactions (such as hydrogen bonding) and covalent interactions (as a certain degree of autoxidation occurred during the incubation process, resulting in the form quinoneauto) [8,9,33,36]. This interaction consequently increased particle size. In the presence of TYRase, the enzyme facilitated covalent interactions between TA and SPI, leading to an additional increase in particle size. A similar increase in particle size was observed when whey protein isolate was covalently modified with rosmarinic acid in the presence of tyrosinase [9].

As expected, the zeta-potential of all samples was negative, consistent with the isoelectric point of SPI, which is below 6.5. Notably, at lower TA concentrations (≤0.01% *w*/*w*), the zeta-potential of modified proteins (SPI-Try, −10.5 mV; SPI-TA1, −10.5 mV; SPI-TA1-Try, −11.1 mV) showed insignificant changes compared to that of the unmodified protein (SPI, −10.8 mV). However, the negative potential value increased with further increased in TA concentration, reaching a peak of −14.6 mV at a TA concentration of 0.05% *w*/*w*. This increase in the negative potential value could be attributed to the neutralization of positively charged groups on SPI by negatively charged quinones formed through TA autooxidation and enzyme-catalyzed oxidation. This neutralization resulted in reduced exposure of positively charged groups and increased exposure of negatively charged groups on the surface of SPI molecules, thereby enhancing the overall negative charge [14].

### 2.2. Intrinsic Fluorescence Spectroscopy Analysis

**Figure 2:**
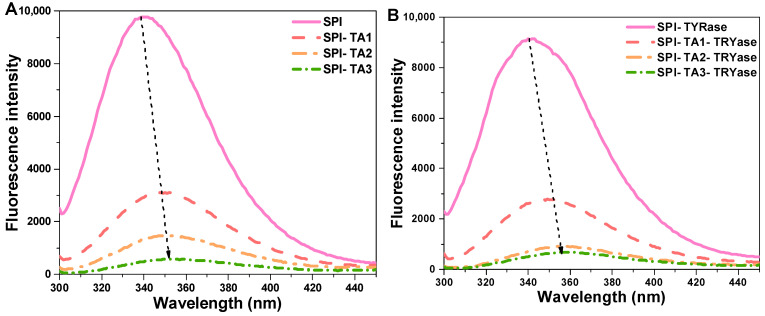
Intrinsic fluorescence spectra of SPI incubated with tannic acid (SPI-TA) (**A**) and SPI incubated with tannic acid and tyrosinase (SPI-TA-TYRase) (**B**) after a 24 h incubation at room temperature (pH 6.5). The dotted arrows in the figure indicated the shift of λ_max_.

Fluorescence spectroscopy, a well-established technique for investigating protein–ligand interactions, was employed to examine the effects of TA modification on the microenvironment surrounding the tryptophan and tyrosine residues in SPI molecules [37]. The fluorescence intensity of SPI incubated with TA alone and incubated with both TA and TYRase was presented in Figure 2. Compared to the control SPI, the maximum fluorescence intensity of all modified SPI decreased, with a more significant reduction observed in the presence of TYRase. These results indicated that the interactions between TA and tryptophan (or tyrosine) residues in SPI molecules were significantly enhanced in the presence of TYRase. This finding was consistent with previous reports on the interactions between zein and quercetagetin [38], α-lactalbumin and caffeic acid [20], as well as lactoferrin and epigallocatechin gallate [39]. The fluorescence intensity of SPI progressively quenched with increasing TA concentration, reaching its minimum at a TA concentration of 0.05% *w*/*w*. These results indicated that the interaction between TA and SPI caused the unfolding of the protein structure and the shielding of the chromophore [40,41]. Additionally, the λ_max_ of SPI-TA samples was gradually red-shifted from 339.2 nm to 354.4 nm as the TA concentration increased (Figure 2A); similarly, the λ_max_ values of SPI-TA-TYRase samples exhibited a significant red-shift from 339.2 nm to 358.6 nm with varying TA concentrations (Figure 2B). The observed red shift in λ_max_ can primarily be attributed to the unfolding of SPI and the exposure of Tyr and Trp residues to a more hydrophilic microenvironment. A similar result was reported in Guo’s study, which demonstrated that covalent bonding between soy protein and tannic acid, induced by alkaline conditions, led to a red-shifted fluorescence [12].

### 2.3. Surface Hydrophobicity Analysis

**Figure 3:**
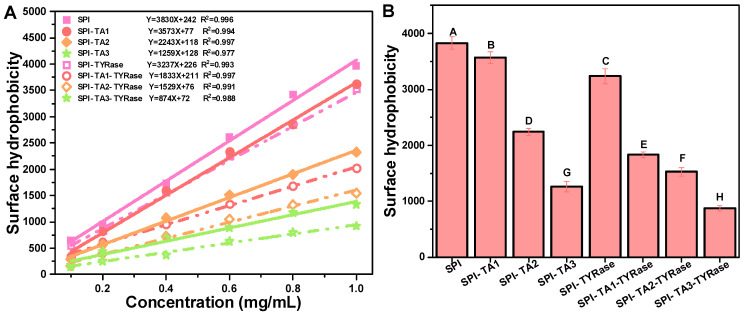
Linear fitting curves and equations of maximum fluorescence intensity at various SPI concentrations (0.1, 0.2, 0.4, 0.6, 08, 1.0 mg/mL) (**A**) and surface hydrophobicity expressed as the initial slope of the linear fitting equation (**B**). Different upper case letters indicate significant differences in the results (*p* < 0.05).

Surface hydrophobicity refers to the specific distribution of hydrophobic groups on the protein surface, which tend to aggregate and significantly influence the functional properties of proteins [40,42]. Measurements of surface hydrophobicity using ANS as a fluorescent probe have been employed to evaluate structural changes in proteins induced by both covalent [9] and non-covalent interactions [43] with other molecules. According to the results obtained from ANS measurements, the R-squared values derived from the linear regression analysis of SPI fluorescence intensity at various concentrations all exceeded 0.97 (Figure 3A). The surface hydrophobicity values of SPI were found to decrease significantly after the binding of TA (*p* < 0.05) regardless of the presence or absence of TYRase (Figure 3B). The surface hydrophobicity of the control SPI was 3830 ± 122. At a TA concentration of 0.05% *w*/*w*, the surface hydrophobicity of modified SPI was measured at 1259 ± 96 (without TYRase) and 874 ± 48 (with TYRase), representing reductions of 67.1% and 77.2% compared to the control SPI, respectively. These results indicated that the exposed surface hydrophobic residues, particularly tyrosine and tryptophan, interacted with TA, leading to a reduction in the overall hydrophobicity of SPI and a consequent decrease in surface hydrophobicity [37,41]. Additionally, the interaction between SPI and TA altered the structural conformation of the protein, exposing previously buried hydrophilic regions and further reducing the surface hydrophobicity of SPI, as evidenced by fluorescence spectroscopy [40]. Meanwhile, TA contains more hydrophilic groups, including hydroxyl and phenolic acid carboxylic groups. These groups were incorporated into protein molecules, thereby enhancing the hydrophilicity of SPI and contributing to the reduction in surface hydrophobicity. These findings were consistent with previous studies that had shown that the reduction in surface hydrophobicity could be attributed to the introduction of polar groups by polyphenols [7,9,11,40]. Incubation with TA and tyrosinase reduced the surface hydrophobicity of SPI, indicating the increase in its surface hydrophilicity. This modification is likely to improve the water holding capacity of SPI, thereby more effectively mimicking the juiciness of animal meat and addressing common issues such as dryness and brittleness in plant-based products.

### 2.4. Fourier Transform Infrared (FTIR) Analysis

**Figure 4:**
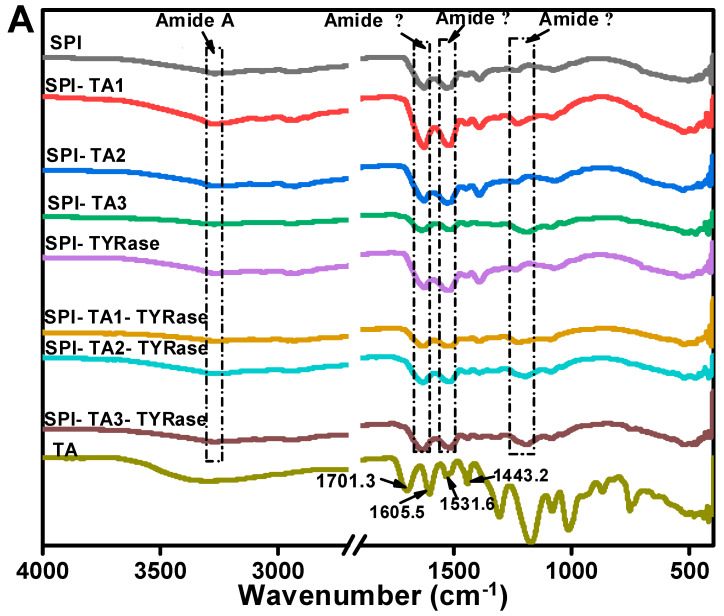

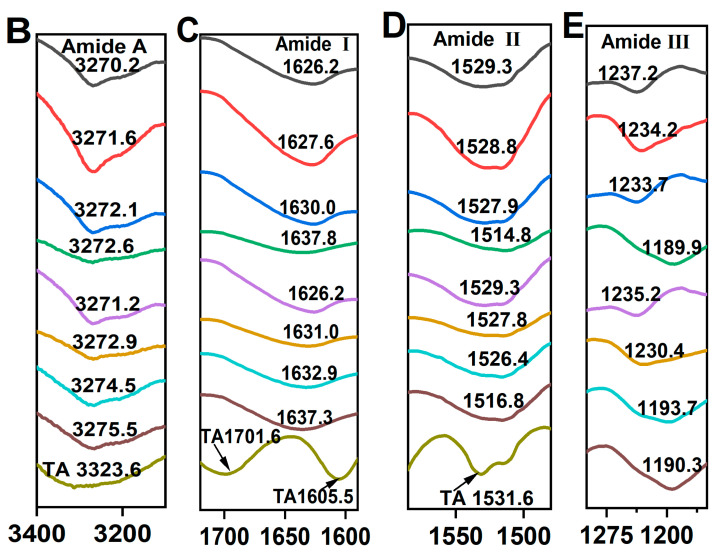
FTIR spectra of SPI incubated with tannic acid (SPI-TA) and SPI incubated with tannic acid and tyrosinase (SPI-TA-TYRase) after a 24 h incubation at room temperature (pH 6.5) (**A**), and the locally magnified image of amide A band (**B**), amide I band (**C**), amide II band (**D**), amide III band (**E**). The samples indicated by different colored lines in (**B**–**E**) were the same as those in (**A**).

The FTIR spectrum of control SPI in Figure 4A, exhibited characteristic amide bands: the amide A band at 3270.2 cm^−1^, attributed to hydrogen bonding and O–H/N–H stretching vibrations; the amide I band at 1625.7 cm^−1^, indicative of C=O stretching and N–H bending; the amide II band at 1529.3 cm^−1^, associated with C–N stretching and N–H bending modes; and the amide III band at 1237.2 cm^−1^, corresponding to side-chain N–H and C–N stretching vibrations [37]. In contrast, the FTIR spectrum of TA exhibited two C=O stretching vibrations at 1701.3 and 1605.5 cm^−1^, a phenolic ring skeletal vibration at 1531.6 cm^−1^ and C=C stretching vibrations of aromatic six-membered rings at 1443.2 cm^−1^ [30]. The peak positions of amide A, I, II, and III bands shifted to varying extents, indicating distinct structural changes in SPI after incubation with TA alone or co-incubation with TA and TYRase. Compared to control SPI, the peak position of SPI-TA3-Tyr near the amide A region exhibited the maximum red-shift from 3270.2 to 3275.5 cm^−1^ (Figure 4B), confirming the participation of the –NH_2_ group of SPI in the reaction. The absence of the absorption band at 3323 cm^−1^, typically attributed to the O–H stretching vibration of TA, in the modified SPI further indicated that phenolic hydroxyl groups were involved in the protein/polyphenol interaction [12]. When polyphenols interact with proteins (both covalent bonding and non-covalent interactions), the stretching and vibration of polyphenols were restricted, leading to the absence of characteristic bands of polyphenols in the spectra of the modified protein molecules, a phenomenon observed in several prior studies [7,17,44]. Upon binding to different concentrations of TA, the amide I band of modified SPI gradually red-shifted from 1626.2 cm^−1^ (control SPI) to 1637.3 cm^−1^ (SPI-TA3-Tyr) (Figure 4C), indicating a significant alteration in the secondary structure of the protein. Additionally, at the highest TA concentration (0.05% *w*/*w*), the amide II and amide III bands of modified SPI blue-shifted to 1516 cm^−1^ and 1190 cm^−1^ (Figure 4D,E), respectively. This suggested that the interaction between SPI and TA induced structural disorder in the SPI molecule [11,41,43], likely due to both nonenzymatic (the autooxidation of TA mentioned above) and enzymatic conjugation between SPI and TA.

### 2.5. Rheological Properties

#### 2.5.1. Apparent Viscosity

**Figure 5:**
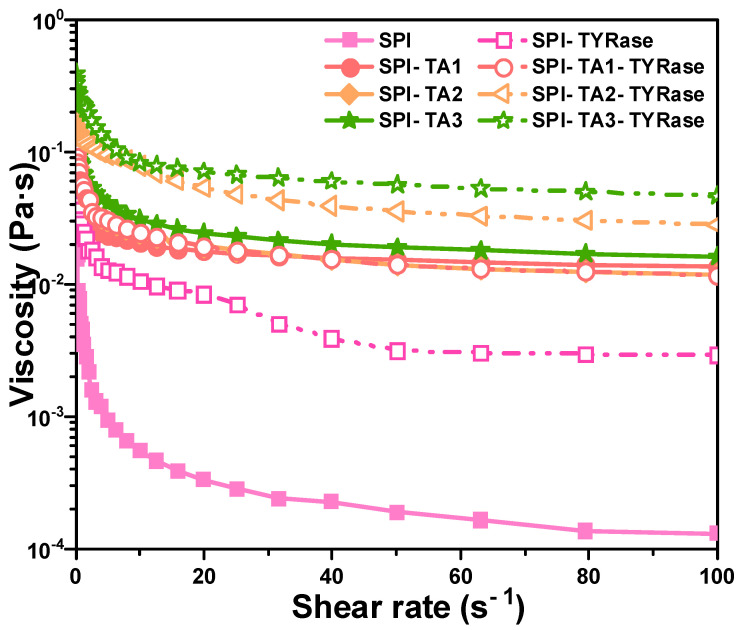
Apparent viscosity curves dependent on shear rate for SPI incubated with tannic acid (SPI-TA) and SPI incubated with tannic acid and tyrosinase (SPI-TA-TYRase) after a 24 h incubation at room temperature (pH 6.5).

The impact of TA concentration on the apparent viscosity of modified protein, both in the presence and absence of TYRase, was examined and compared, as illustrated in Figure 5. The results demonstrated that the apparent viscosity of both control SPI and modified SPI samples decreased with an increase in shear rate, indicating significant shear-thinning behavior, consistent with previous studies on the rheology of SPI solution [45]. Shear-thinning behavior had been previously observed in food hydrophilic colloid systems, which can be attributed to substantial shear-induced structural breakdown. Additionally, within the shear rate range of 0.1–100 s^−1^, the apparent viscosity exhibited a positive correlation with increasing TA concentration. Notably, the apparent viscosity of SPI-TA-TYRase samples was consistently higher than that of SPI-TA samples. This phenomenon could be attributed to four types of interactions present in SPI-TA-TYRase samples: (1) non-covalent interactions, such as hydrogen bond and hydrophobic bond between SPI and TA [10]; (2) covalent cross-linking complexes formed when the quinone produced by the autooxidation of TA reacted with the amino groups of SPI [9]; (3) TYRase-catalyzed conversion of TA into quinone in the presence of oxygen, followed by amino addition reactions with SPI [9]; and (4) TYRase-catalyzed formation of quinones from tyrosine residues within SPI molecules, leading to covalent cross-linking within or between SPI molecules [46]. In contrast, only the first two interactions were present in SPI-TA samples, resulting in a lower viscosity compared to SPI-TA-TYRase.

#### 2.5.2. Viscoelastic Properties

**Figure 6:**
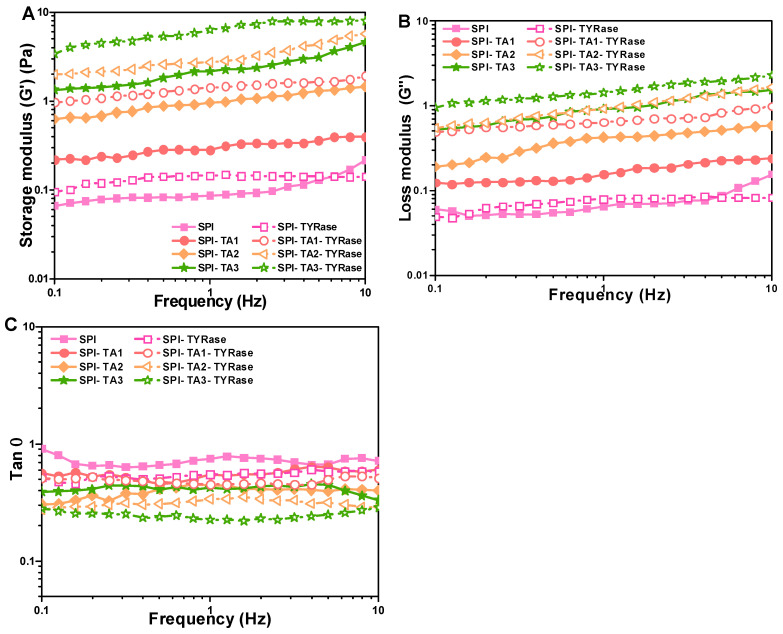
Changes in storage modulus (G′) (**A**), loss modulus (G″) (**B**), and loss factor (tan) (**C**) dependent on frequency for SPI incubated with tannic acid (SPI-TA) and SPI incubated with tannic acid and tyrosinase (SPI-TA-TYRase). The tests were conducted at 25 °C.

Figure 6A,B illustrated the variation in storage (G′) and loss (G″) modulus values with frequency for samples containing different concentrations of TA. The G′ was consistently higher than G″ (G′ > G″), indicating that elastic behavior dominated the system and suggesting gel-like behavior [47]. Furthermore, both G′ and G″ increased with higher concentrations of TA, suggesting non-covalent and covalent interactions enhanced. Notably, the G′ and G″ values for SPI incubated with both TA and TYRase were significantly higher compared to those incubated with TA alone, indicating enhanced covalent crosslinking. In the section of apparent viscosity, similar results were reported: the SPI-TA-TYRase samples exhibited greater intramolecular and intermolecular cross-linking strength due to the presence of four cross-linking modes, in contrast to the two cross-linking modes present in the SPI-TA samples. Additionally, the loss factor (tan δ, obtained from G″/G′), provided insights into the balance between the viscoelastic moduli of the protein network [42]. A lower tan δ signified a greater contribution from the elastic component. As anticipated in food products, a more pronounced elastic component enhanced the stiffness of the gel-like structure [47]. As depicted in Figure 6C, the control SPI exhibited the highest tan δ, coupled with a low storage modulus across the frequency range of 0.1–10 Hz, indicating a relatively weak gel-like structure or even solution behavior, characterized by a greater contribution from the viscous component. The co-incubation of TA and TYRase significantly reduced the tan δ values of the samples, with the lowest tan δ observed for the SPI-TA3-TYRase sample. This indicated a substantial improvement in elasticity and a more gel-like structure due to the increased formation of covalent bonds, consistent with previous observations in enzymatically crosslinked protein gels [16,42], and protein-stabilized emulsion gels [47,48]. These results indicated that modified SPI could positively influence dough structure in baking applications. For instance, the increased storage modulus (G′) and loss modulus (G″) observed suggest improved gas retention and dough elasticity, which are critical for leavened products such as bread or cakes. Additionally, SPI-polyphenol interactions could reduce starch retrogradation, delaying staling and extending product freshness.

### 2.6. Protein Microstructure

**Figure 7:**
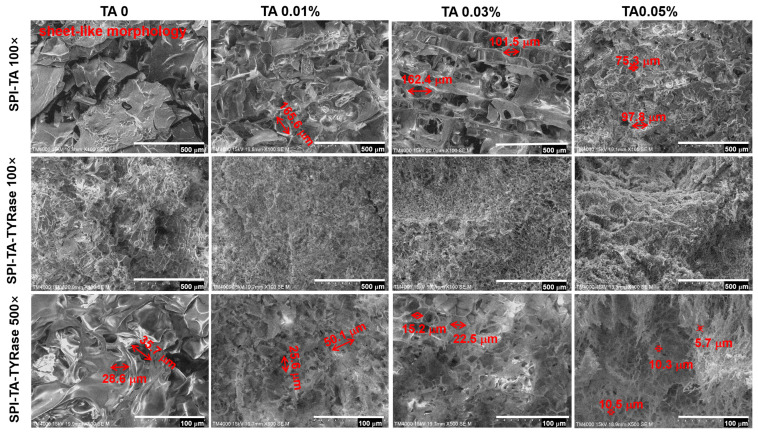
Scanning electron microscopy (SEM) images of SPI, SPI incubated with tannic acid (SPI-TA), and SPI incubated with tannic acid and tyrosinase (SPI-TA-TYRase) at magnifications of 100× and 500×.

The microstructure of the control SPI and the modified SPI incubated with only TA and with both TA and TYRase was presented in Figure 7. In contrast to the sheet-like morphology of the control sample, the SPI-TA samples exhibited a pronounced network structure. As the concentration of TA increased, the initially large and loosely distributed pores gradually became smaller (from 165.6 μm to 75.3 μm) and more densely packed. Furthermore, the SPI-TA-TYRase samples revealed a dense and uniform network structure. At 500× magnification (scale bar: 100 μm), it was evident that the microstructure of the SPI-TA-TYRase samples displayed dense rectangular or polygonal porosity. As the concentration of TA increased, both pore density and the reduction in pore size (from 35.7 μm to 5.7 μm) became more pronounced, correlating with the observed trends in viscosity and G′ values from the rheological analysis. The microstructural alterations in SPI-TA and SPI-TA-TYRase compared to control SPI, can be attributed to both non-covalent and covalent interactions between polyphenols and proteins, which led to changes in chemical bonding and consequently modified the microstructure. This observation aligns with the microstructural characteristics reported in previous research [12,35,49].

## 3. Conclusions

TYRase-catalyzed aggregates of SPI with TA were fabricated and the structural, rheological properties and microstructure were systematically investigated in comparison to control samples of SPI and SPI incubated with TA alone. Particle size and polydispersity index (PDI) analysis confirmed that TYRase-induced crosslinking with TA through covalent binding resulted in SPI aggregation, which was positively correlated with TA concentration. Following covalent binding with TA, the structure of SPI underwent significant changes, as evidenced by the gradual red shift in the amide I band and the blue shifts in the amide II and amide III bands. Furthermore, as the concentration of TA increased, the fluorescence intensity decreased markedly, indicating that the microenvironment surrounding aromatic amino acids became more hydrophilic. The surface hydrophobicity of SPI also decreased significantly with increasing TA concentration. Both the storage modulus (G′) and loss modulus (G″) of SPI incubated with TA and TYRase were significantly enhanced, with G′ > G″, indicating typical gel-like behavior. The denser and finer pore network structure further confirmed that the gel-like properties of SPI-TA-TYRase samples were effectively improved, likely due to the covalent crosslinking between SPI and TA facilitated by TYRase. The findings highlight the potential of this approach for improving protein functionality in food systems, such as meat analogs and baking applications. Overall, this study demonstrates that tyrosinase-catalyzed crosslinking with tannic acid significantly enhances the structural and functional properties of SPI. However, there may be some limitations to the application of this modification in the food industry, such as differences in enzyme activity from batch to batch, enzyme odor, processing temperature and pH levels. Therefore, future research will focus on scaling up the modification process, optimizing conditions for industrial applications, and evaluating the sensory and nutritional properties of SPI-TA complexes.

## 4. Materials and Methods

### 4.1. Materials

Powdered soybean protein isolate (SPI, dry basis content 99%) was obtained from Shanghai YI EN Chemical Technology Co. (Shanghai, China). Tannic acid (TA, 98%; Lot #D2114023) was procured from Shanghai Aladdin Biochemical Technology Co., Ltd. (Shanghai, China). Tyrosinase (TYRase, derived from mushroom, 1136 U/mg) was acquired from Shanghai Yuanye BioTechnology Co., Ltd. (Shanghai, China). All other reagents used in this study were of analytically pure grade. All samples were prepared using double-distilled water produced by a laboratory-grade water purification system.

### 4.2. Sample Preparation

Powdered SPI (10%, *w*/*w*) was dispersed in distilled water with constant stirring (600 rpm) at 25 °C for 2 h, followed by a full hydration for 12 h at 4 °C to create stock solutions. To minimize TA autooxidation, a fresh TA stock solution (1.0% *w*/*w*) was prepared immediately before use.

The SPI and SPI-TA mixed solutions were generated by diluting the SPI stock solution (with a final concentration fixed at 3% *w*/*w*) and incorporating varying levels of TA (0, 0.01, 0.03, and 0.05% *w*/*w*), with the pH adjusted to 6.5 using 2 M NaOH.

Subsequently, SPI-TA solutions with TYRase at a final concentration of 80 U/g protein were incubated at 25 °C. The amount of enzyme added was referred to the method of Yuan et al. [50]. The pH of the system was monitored throughout the incubation and maintained at 6.5. After 24 h incubation, the sample was heated for 15 min at 60 °C to inhibit TYRase activity, then cooled using an ice water bath and stored at 4 °C or freeze-dried for subsequent analysis. The SPI and SPI-TA samples incubated without TYRase under the same conditions served as controls.

The samples, characterized by progressively increasing TA concentrations (0, 0.01, 0.03, 0.05% *w*/*w*), are categorized into two groups: the group incubated with TA alone (SPI, SPI-TA1, SPI-TA2, SPI-TA3) and the group incubated with both TA and TYRase (SPI-TYRase, SPI-TA1-TYRase, SPI-TA2-TYRase, SPI-TA3-TYRase).

In our study, increasing the amount of TA beyond 0.05% could result in phase separation. TA-SPI complexes were soluble at TA concentrations less than 0.05%, as confirmed by dynamic light scattering analysis (PDI < 1.0). Therefore, all experiments were conducted at TA concentration less than 0.05%.

### 4.3. Particle Size and Zeta-Potential Measurement

Samples of SPI, SPI-TA, and SPI-TA-TYRase were diluted to a 0.5% protein concentration using PBS (10 mM, pH 6.5). The particle size and zeta-potential were measured with a Malvern Zetasizer Nano ZS (Malvern Instruments, Malvern, UK) at 25 °C. The particle refractive indices used were 1.45 for SPI and 1.33 for PBS (10 mM, pH 6.5).

### 4.4. Intrinsic Fluorescence Spectroscopy Measurement

The intrinsic fluorescence spectra of the samples were recorded using a spectrofluorimeter (F-4600, Hitachi, Tokyo, Japan) at 25 °C. Final protein concentrations were adjusted to 0.2 mg/mL in PBS (10 mM, pH 6.5). An excitation wavelength of 290 nm was employed to monitor the changes in the microenvironment of aromatic amino acids. Emission was recorded for wavelengths between 300 and 450 nm, with the background of PBS subtracted from the respective samples spectra [51]. Both excitation and emission slits were set to 5 nm, while the scanning voltage and speed were maintained at 600 V and 1200 nm/min, respectively.

### 4.5. Surface Hydrophobicity Measurement

The surface hydrophobicity of the modified SPI was determined using the method established by Wang et al. [40], employing a fluorescence spectrometer (F-4600, Hitachi, Tokyo, Japan). The 1-anilino-8-naphthalensulfonate (ANS) served as the fluorescence probe. In summary, six different protein concentrations ranging from 0.1 to 1.0 mg/mL in PBS (10 mM, pH 6.5,) were prepared for each sample. Subsequently, 50 μL of 8.0 mM ANS stock solution (10 mM, pH 6.5, PBS) was mixed with each sample (10 mL), and the mixture was kept in the dark for 20 min at room temperature. The fluorescence intensity was measured at an excitation wavelength of 390 nm and an emission wavelength of 470 nm. The blank sample, which contained only the buffer and ANS, was also prepared and its readings were subtracted from all experimental measurements. The surface hydrophobicity of each sample was calculated and expressed as the initial slope of the maximum fluorescence intensity versus protein concentration, determined through linear fitting analysis.

### 4.6. Fourier Transform Infrared (FTIR) Measurement

FTIR spectra were obtained by scanning from 4000 cm^−1^ to 400 cm^−1^ at a resolution of 4 cm^−1^ with 32 scans per sample using a Jasco 4100 series spectrometer (Jasco Inc., Easton, PA, USA). Prior to scanning, freeze-dried samples were mixed with KBr powder at a ratio of 1:50 (*w*/*w*) and compressed into thin circular pellets. A blank KBr pellet was scanned as a background reference before each sample measurement. Spectra were baseline-corrected before interpretation.

### 4.7. Rheological Properties Measurement

The rheological properties of SPI-TA and SPI-TA-TYRase samples were determined using a Kinexus Lab + rheometer (Malvern Instruments, Malvern, UK) at 25 °C. After loading the sample, a two-minute period was allowed for the sample to return to its original state. The viscosity of the sample was recorded over a shear rate range of 0.1 to 100 s^−1^. The frequency sweeping was performed at 0.3% strain, and the storage modulus (G′) and loss modulus (G″) were recorded over a frequency ranged of 0.1 to 10 Hz.

### 4.8. Microstructure Observation

The surface microstructure of the samples was characterized using a tabletop scanning electron microscope (TM4000Plus, Hitachi Scientific Instruments Co. LTD, Beijing, China) operated at an acceleration voltage of 15.0 kV and magnifications of 100× and 500×. The samples were fixed onto the sample table with double conductive tape under vacuum for analysis.

### 4.9. Statistical Analysis

All experiments were conducted in triplicate, and the results are expressed as means ± standard deviations (SD). Data and figures were processed using WPS Office 2024 and Origin 2021. Differences between samples were calculated using the Least Significant Difference (LSD) method (SPSS Statistics 22.0, *p* < 0.05). Prior to applying the LSD test, the normality of the data distribution and homogeneity of variances were rigorously tested to validate the assumptions underlying the statistical analysis. All experiments were performed in triplicate, and the values were reported as the mean ± standard deviation.

## Data Availability

The original contributions presented in the study are included in the article, further inquiries can be directed to the corresponding author.

## Abbreviations

The following abbreviations are used in this manuscript:

SPI	Soy protein isolate
TA	Tannic acid
TYRase	Tyrosinase
PDI	Polydispersity index
G′	Storage modulus
G″	Loss modulus
